# Supplementary material for: Distinct activation mechanisms of β-arrestin-1 revealed by 19F NMR spectroscopy
Source: Nat Commun. 2023 Nov 29;14:7865. doi: 10.1038/s41467-023-43694-1 (PMC10686989; doi:10.1038/s41467-023-43694-1)
Supplement: Supplementary file 1 — Supplementary Information [file 41467_2023_43694_MOESM1_ESM.pdf]

Supplementary Information for "Distinct activation mechanisms of β-arrestin 1 revealed by ^{19}F NMR spectroscopy"

Ruibo Zhai, Zhuoqi Wang, Zhaofei Chai, Xiaogang Niu, Conggang Li, Changwen Jin, Yunfei Hu

This file contains Supplementary Figures 1-13, Supplementary Table 1-4, Supplementary Discussion and Supplementary References.

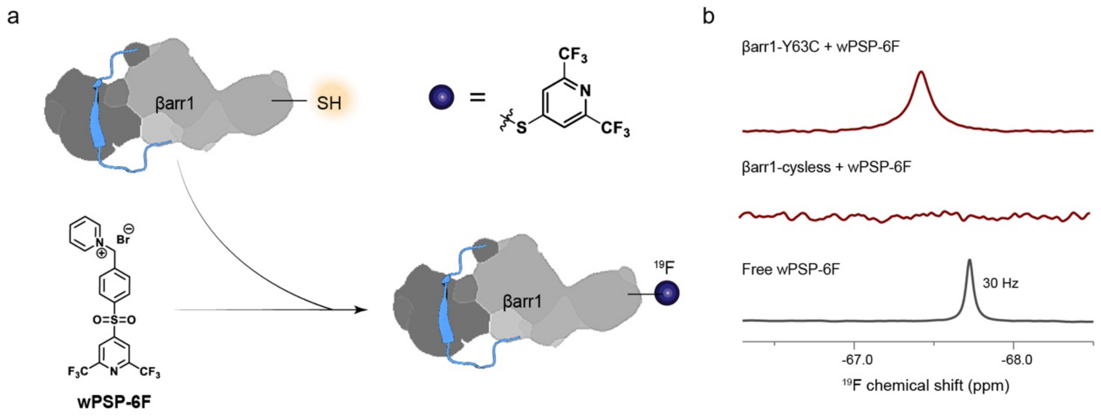

c

1	MGDKGTRVFK	KASPNGKLTV	YLGKRDFVDH	IDLVDPVDGV	VLVDPEYLKE	50
	RRVYVTLTVA	FRYGREDLDV	LGLTFRKDLF	VANVQSFPPA	PEDKKPLTRL	100
N domain	QERLIKKLGE	HAYPFTFEIP	PNLPSSVTLQ	PGPEDTGKAL	GVDYEVKAFV	150
	AENLEEKIHK	RNSVRLVIRK	VQYAPERPGP	QPTAETTRQF	LMSDKPLHLE	200
	ASLDKEIYYH	GEPISVNVHV	TNNTNKTVKK	IKISVRQYAD	IVLFNTAQYK	250
C domain	VPVAMEEADD	TVAPSSTFSK	VYTLTPFLAN	NREKRGLALD	GKLKHEDTNL	300
	ASSTLLREGA	NREILGIIVS	YKVKVKLVVS	RGGLLGDLAS	SDVAVELPFT	350
	LMHPKPKEEP	PHREVPENET	PVDTNLIELD	TNDDDIVFED	FARQRLKGMK	400
C tail	DDKEEEEDGT	GSPQLNNR				418

Supplementary Fig. 1. ^{19}F-labeling scheme for βarr1. (**a**) A schematic illustration showing the single-site labeling of the wPSP-6F ^{19}F-probe [1] by reaction with a free cysteine thiol group. (**b**) Representative spectra of βarr1 showing the verification of successful ^{19}F labeling, and comparison with the ^{19}F spectrum of the free probe. (**c**) Amino acid sequence of the full-length wild-type βarr1 protein with ^{19}F-labeling sites highlighted.

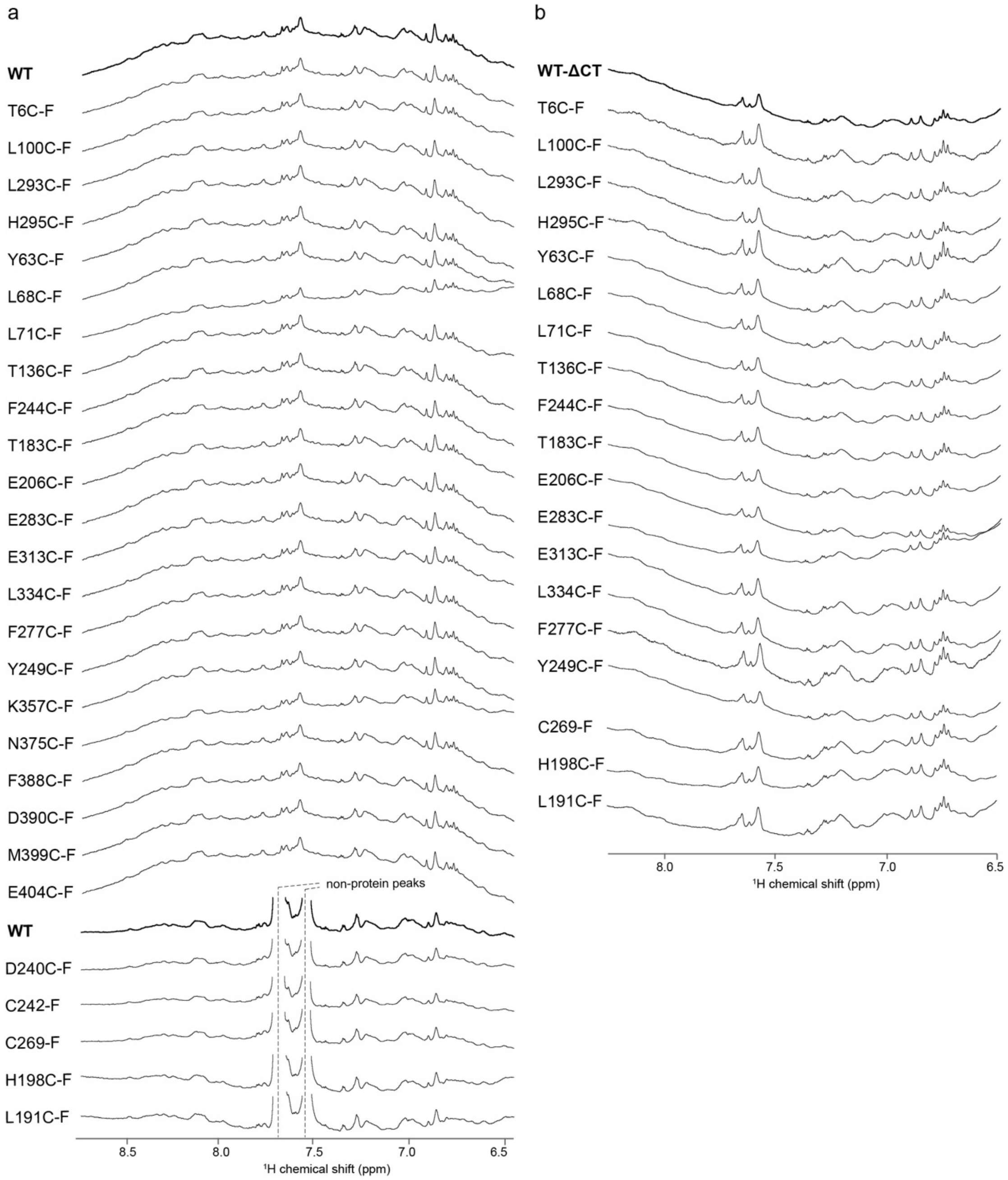

Supplementary Fig. 2. ^{1}H NMR verification of the structural integrity of the ^{19}F-labeled βarr1 samples. (**a**) ^{1}H NMR spectra of the ^{19}F-labeled βarr1 samples compared with the wild-type full-length protein showing the amide signal region. The five ^{19}F-labeled samples shown at the bottom (D240C, C242, C269, H198C and L191C) were prepared using a different batch of buffer in which cocktail protease inhibitor, TFA and DTT were pre-added, and was therefore compared with a WT sample using the same buffer. The strong peaks in the 7.5-7.7 ppm region originate from the small molecules. (**b**) ^{1}H NMR spectra of the ^{19}F-labeled βarr1-ΔCT samples compared with the wild-type βarr1-ΔCT showing the amide signal region.

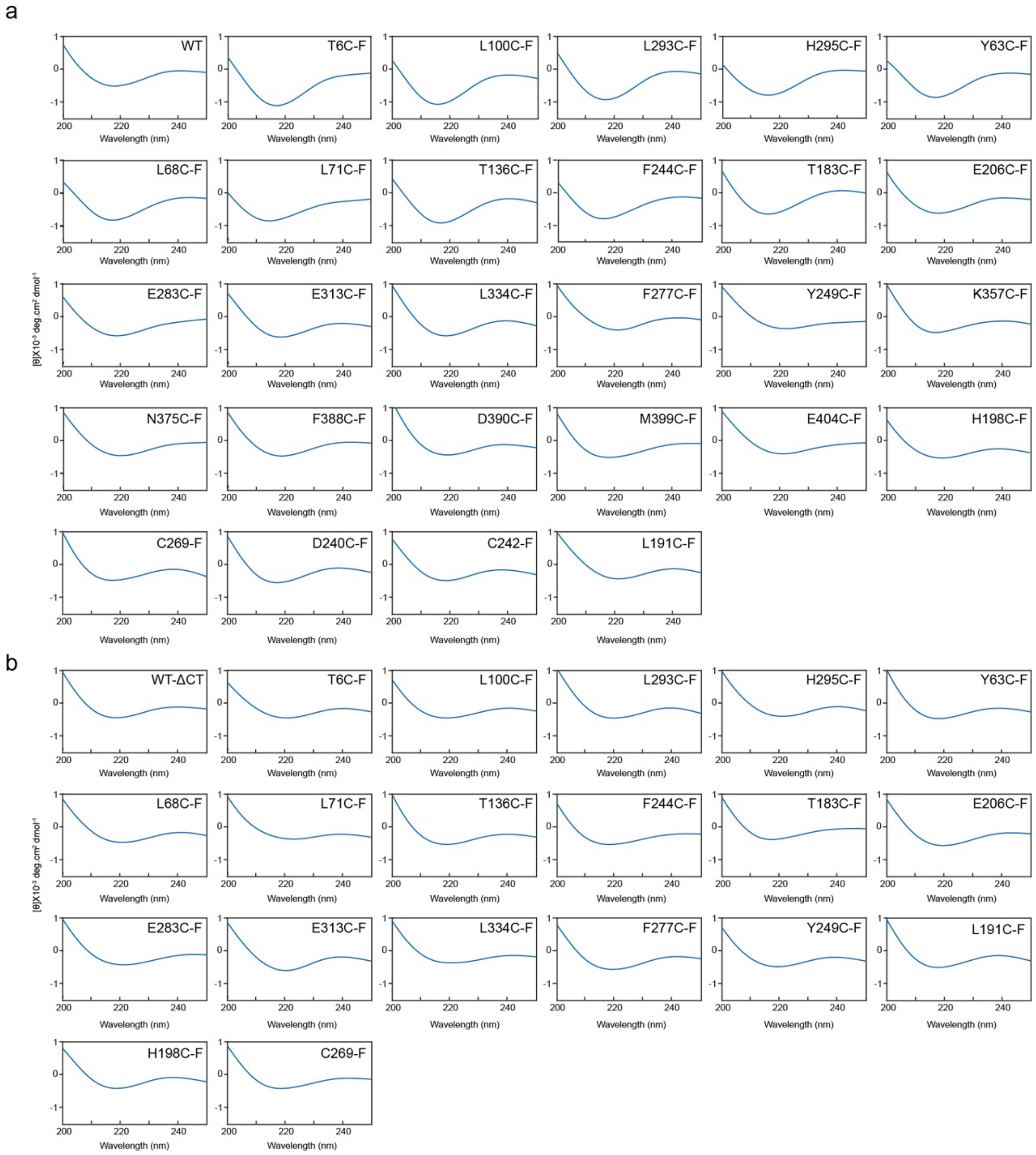

Supplementary Fig. 3. CD spectra verification of the structural integrity of the ^{19}F-labeled βarr1 samples. (**a**) CD spectra of the ^{19}F-labeled βarr1 samples compared with the wild-type full-length protein. (**b**) CD spectra of the ^{19}F-labeled βarr1-ΔCT samples compared with the wild-type βarr1-ΔCT protein.

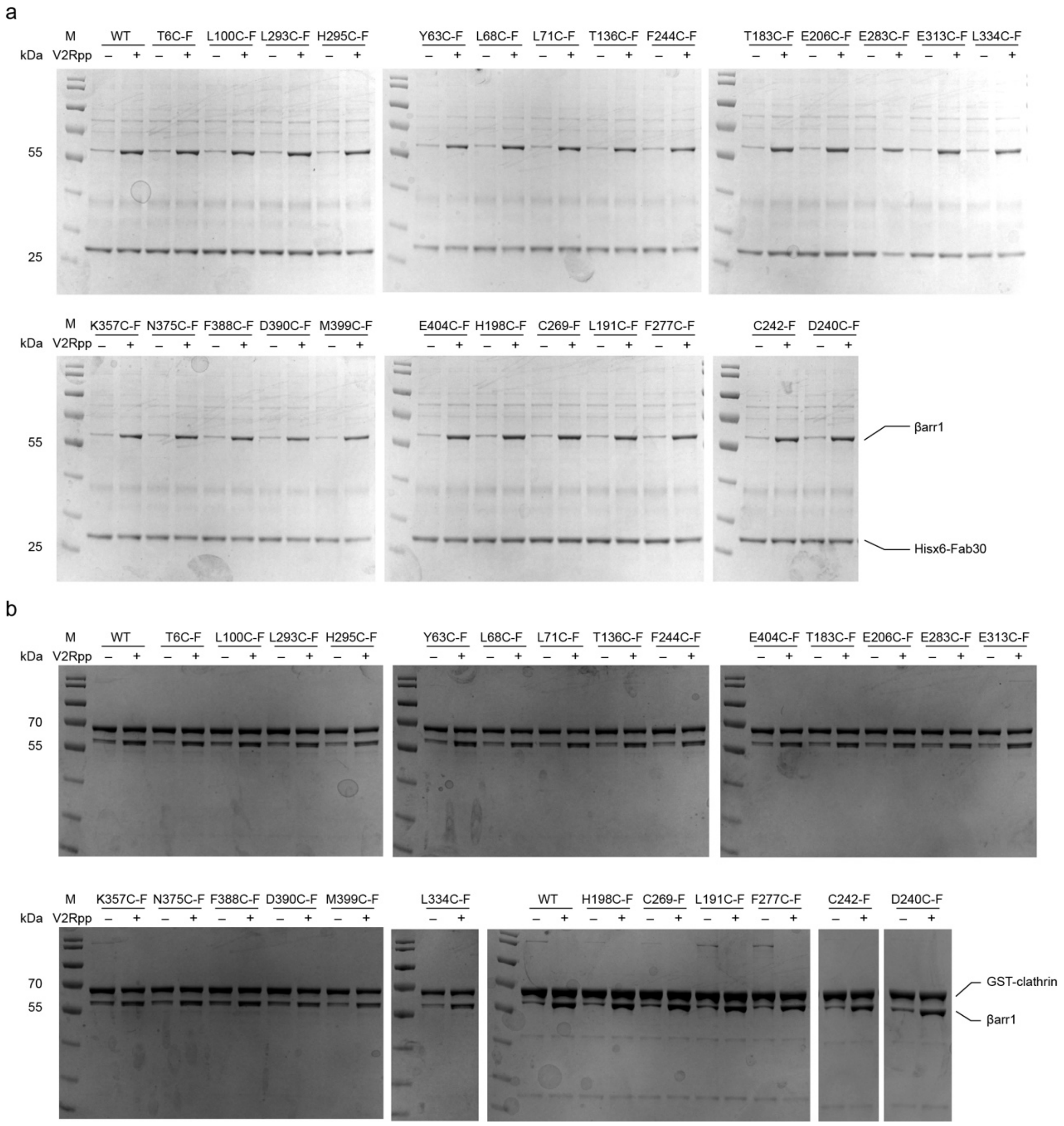

Supplementary Fig. 4. Functional verifications of the ^{19}F-labeled βarr1 samples. (**a**) Ni-affinity pull-down assays showing the V2Rpp-enhanced binding to Fab30 by the wild-type and ^{19}F-labeled βarr1 proteins. (**b**) GST pull-down assays showing the V2Rpp-enhanced binding to clathrin by wild-type and ^{19}F-labeled βarr1 proteins.

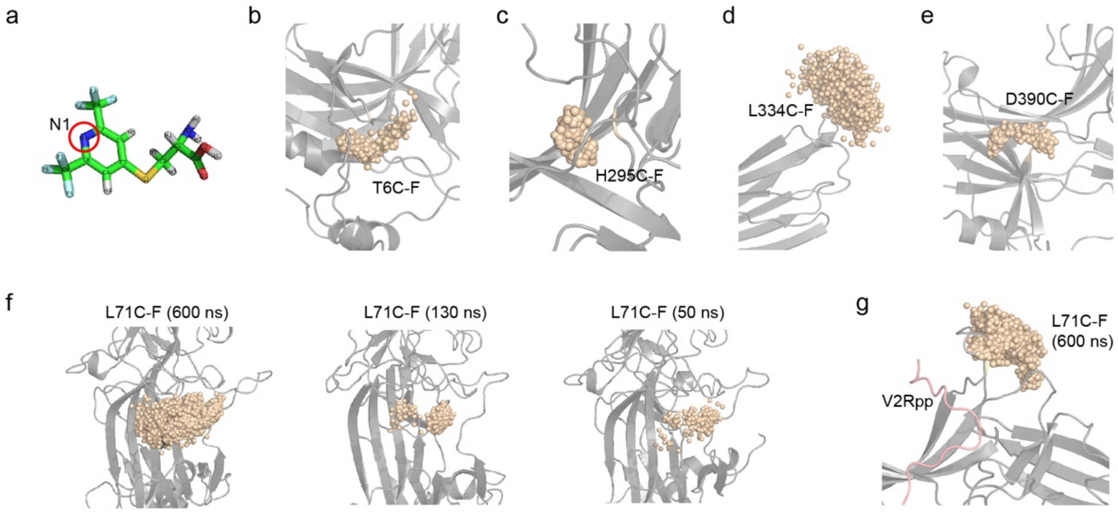

Supplementary Fig. 5. Molecular dynamics simulations of ^{19}F-labeled βarr1. (**a**) Chemical structure of the wPSP-6F probe ligated to a cysteine residue. The N1 atom is indicated. (**b-e**) MD simulation results showing the spatial distributions of the N1 atom of the probe in all simulation frames in a 200 ns simulation obtained for the T6C, H295C, L334C and D390C-labeled βarr1 in the basal state. (**f**) Spatial distributions of the N1 atom of the probe obtained for the L71C-labeled βarr1 in the basal state in three simulation trajectories lasting 600 ns, 130 ns and 50 ns. (**g**) Spatial distributions of the N1 atom of the probe obtained for the L71C-labeled βarr1 in the V2Rpp-bound state in a 600 ns simulation.

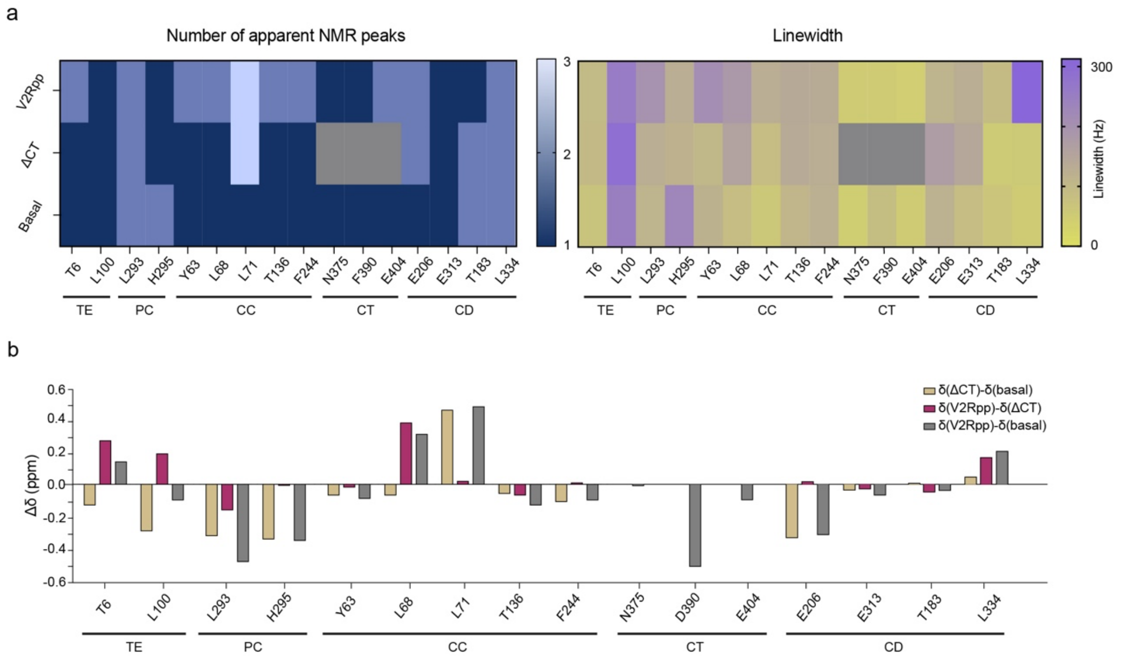

Supplementary Fig. 6. V2Rpp-induced conformational changes in critical regions of βarr1. (**a**) Summary of the apparent number of NMR peaks (left) and linewidth of NMR peaks (right) in key structural regions in the basal, ΔCT and V2Rpp-bound states of βarr1. (**b**) Chemical shift changes for representing residues in key structural regions in the basal, ΔCT and V2Rpp-bound states of βarr1. The TE, PC, CC, CT abbreviations stand for the three-element interacting site, the polar core, the central crest and the carboxyl tail, respectively. For residues showing multiple peaks, only the linewidth of the major peak is shown in (**a**) and the largest chemical shift change is presented in (**b**). Grey color indicates the absence of data.

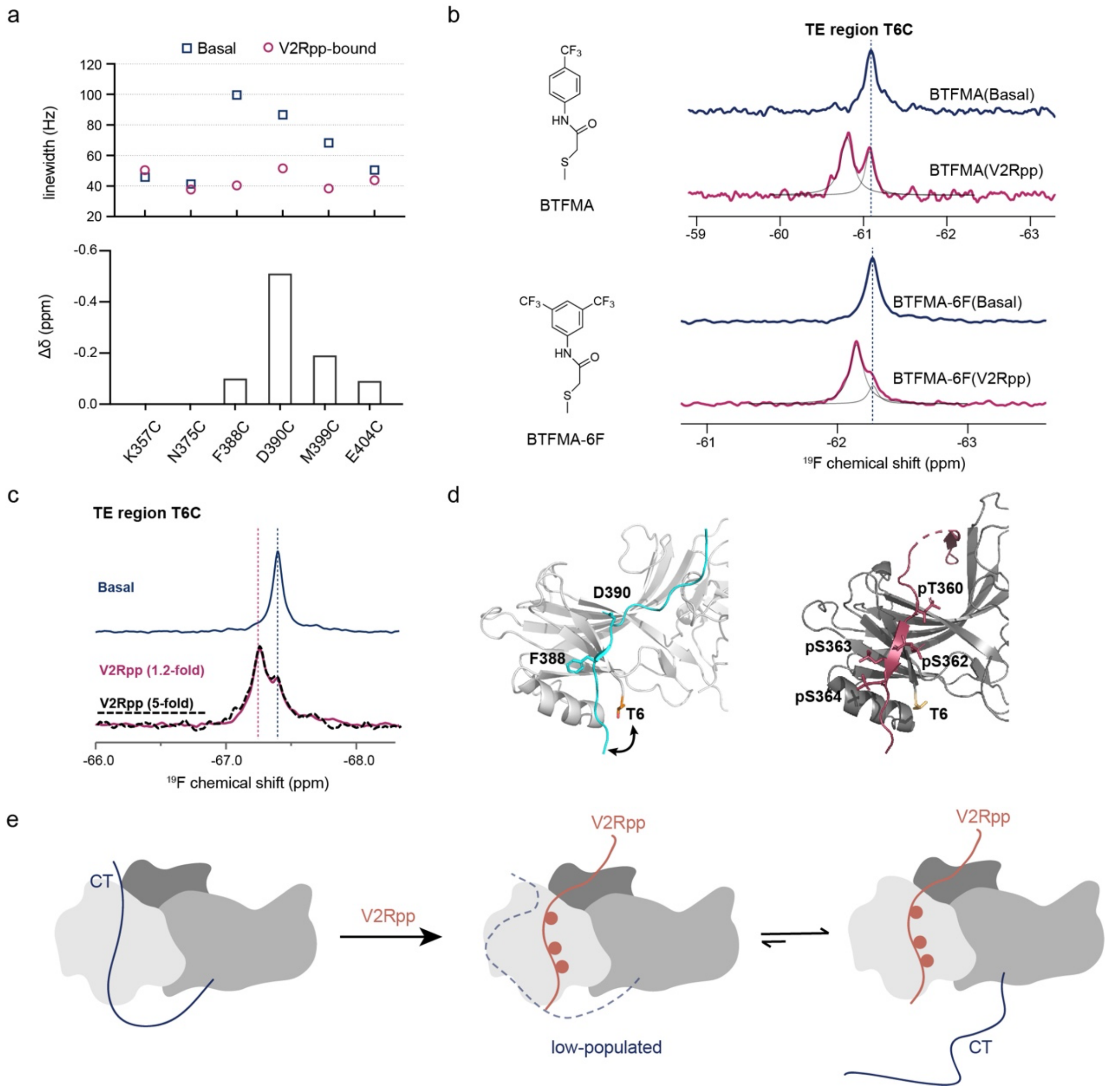

Supplementary Fig. 7. V2Rpp-induced changes in the CT and TE regions. (**a**) Linewidths and chemical shift differences of the CT region in the basal and V2Rpp-bound states. (**b**) Verification of the dual resonances observed at the T6C site upon V2Rpp binding by using different ^{19}F probes. The chemical structure of the BTFMA and BTFMA-6F probes are shown. (**c**) ^{19}F NMR spectra of the wPSP-6F-labeled T6C site titrated with 1.2 or 5-fold excess of V2Rpp compared to the basal state. (**d**) Comparison of the local structure at the TE region in the basal (left) and V2Rpp-bound (right) states showing the location of residue T6. The side chains of F388 and D390 in the basal state and the cluster of phosphates in the peptide in the V2Rpp-bound state are shown as sticks. (**e**) A schematic illustration showing CT and TE region conformational dynamics in the V2Rpp-bound state. Both the distal CT and the proximal CT region close to the TE site may comprise a subpopulation that remains contact with the N-domain in the presence of V2Rpp.

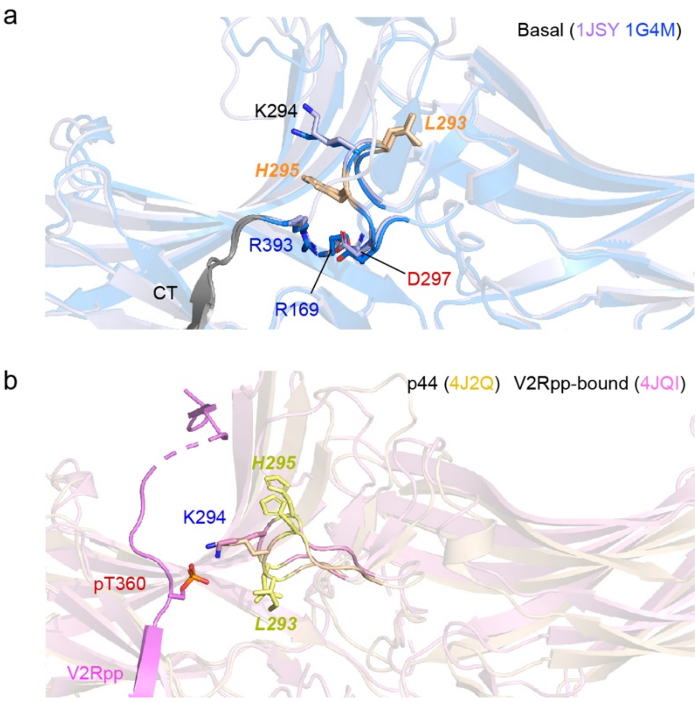

Supplementary Fig. 8. Local structure of the gate loop observed in crystal structures. (**a**) Gate loop conformations observed in the inactive state crystal structures, including PDB 1JSY and 1G4M. (**b**) Gate loop conformations observed in the V2Rpp-bound active state crystal structure (PDB: 4JQI) and the CT-truncated splice variant p44 of visual arrestin (PDB 4J2Q).

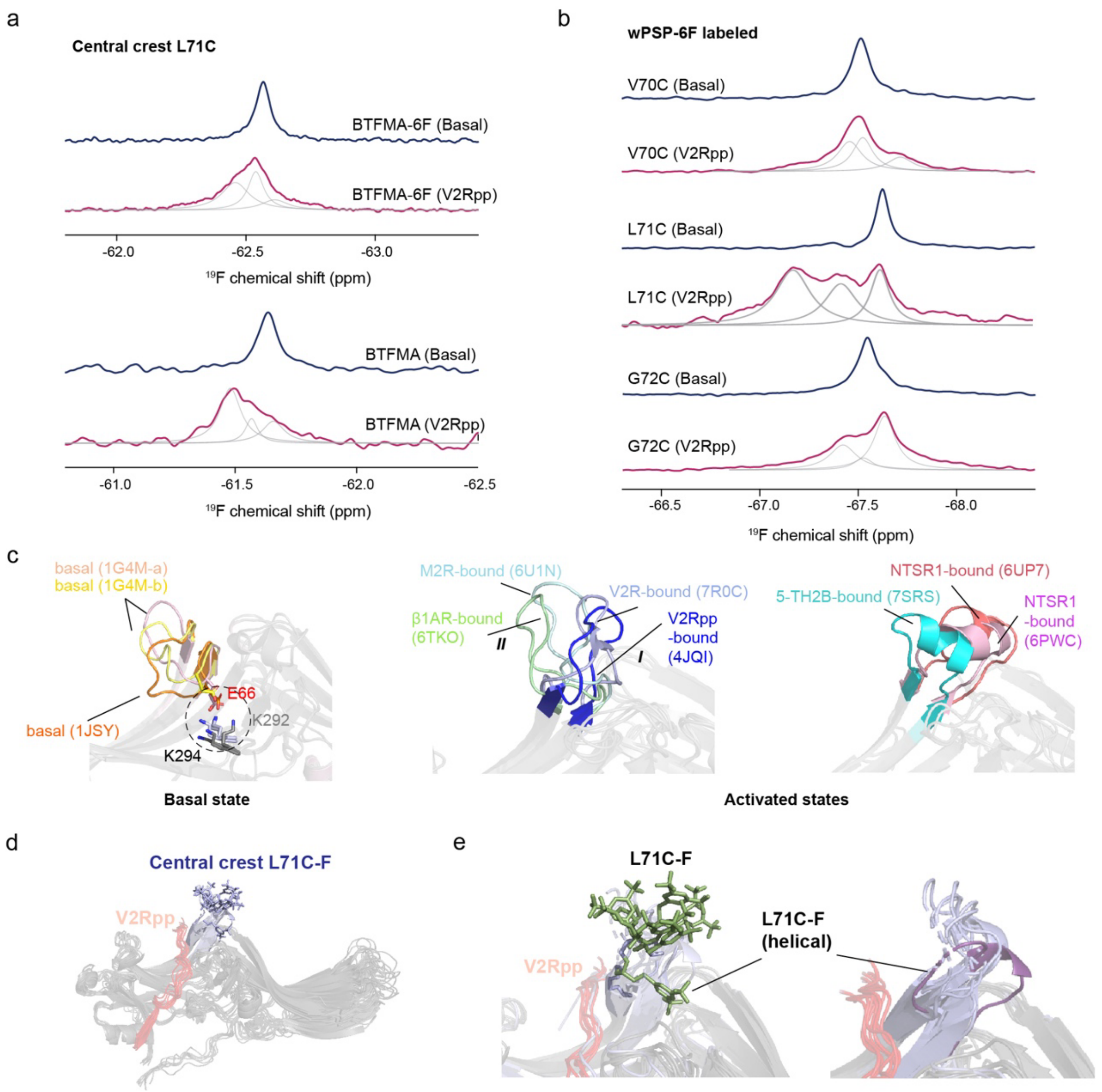

Supplementary Fig. 9. Dynamics of the finger loop in the V2Rpp-bound state. (**a**) Verification of the multiple resonances observed at the L71C site upon V2Rpp binding by using different ^{19}F probes BTFMA and BTFMA-6F. (**b**) ^{19}F NMR spectra of the wPSP-6F-labeled V70C, L71C and G72C sites in the basal and V2Rpp-bound states. Peak deconvolutions are shown as light grey lines in (**a**) and (**b**). (**c**) Different conformations of the finger loop observed in the crystal or cryo-EM structures. The L71 residue is shown in sticks in all structures. (**d-e**) MD simulation results of the wPSP-6F-labeled L71C mutant in the V2Rpp-bound state showing the full structure (**d**) and the finger loop region (**e**). The local helical-like conformation observed during the simulation is indicated.

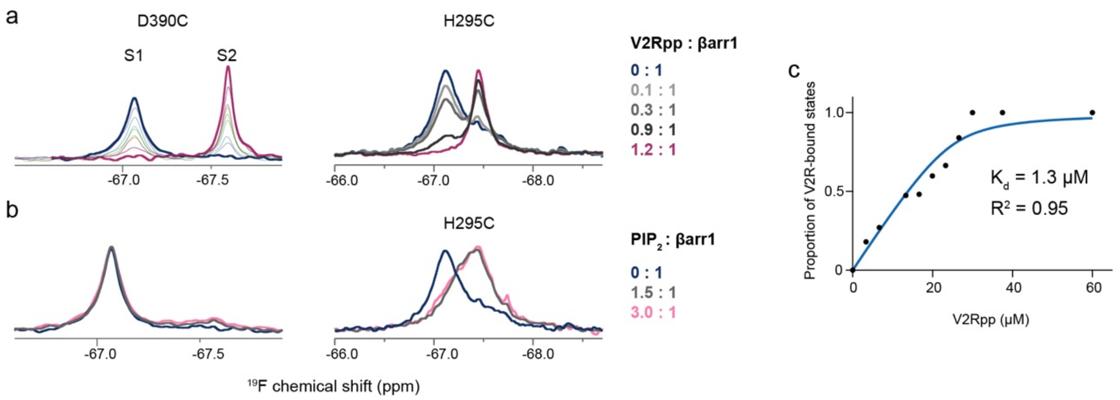

Supplementary Fig. 10. Titration experiments of V2Rpp or PIP_2 into βarr1 probed by ^{19}F NMR. (**a**) Titration of V2Rpp up to 1.2:1 molar ratio fully activates βarr1 at both D390C and H295C sites. (**b**) Titration of PIP_2 up to 3.0:1 molar ratio partially activates βarr1 at the H295C site, but minimally affects the D390C site. (**c**) Estimation of the V2Rpp-βarr1 binding affinity based on peak intensity changes of the D390C site of βarr1 titrated with V2Rpp. The data were fitted using the equation $\theta = \frac{(K_d+P+L)-\sqrt{(K_d+P+L)^2-4PL}}{2P}$ [2], where K_d is the dissociation constant, P is the total concentration of the βarr1 protein, L is the total concentration of the V2Rpp peptide, and θ is the fraction of bound state of βarr1. The values of θ were calculated as by $\theta = V_{S2}/(V_{S1} + V_{S2})$, in which V_{S1} and V_{S2} correspond to the volume integrals of the peaks of S1 and S2 states, respectively. The data were best fitted with K_d of ~1.3 μM with correlation coefficient R^2 = 0.95. Because the NMR titration experiments were conducted at protein and peptide concentrations much larger than the K_d itself, the fitting result may not be accurate and should only be regarded as an estimation.

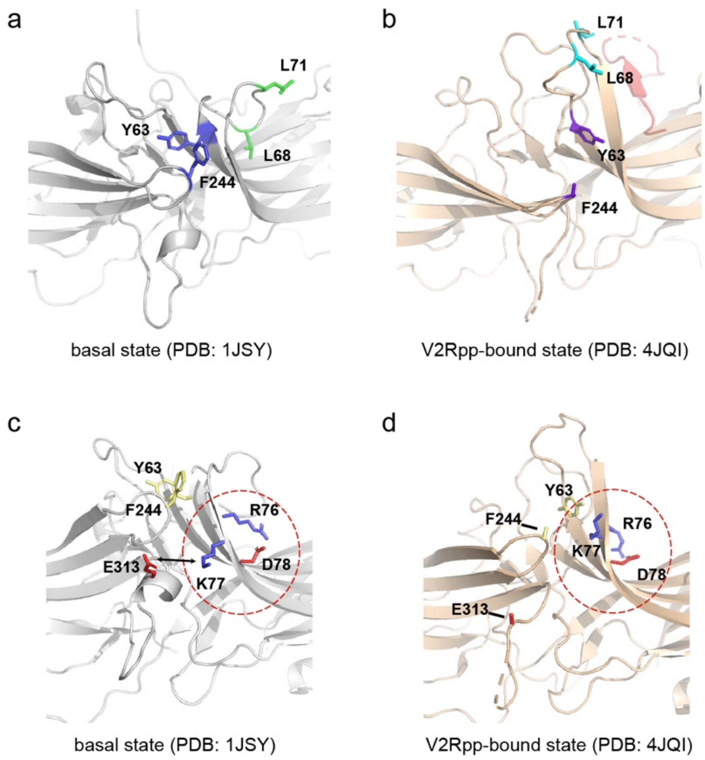

Supplementary Fig. 11. Local conformations at the back side of the βarr1 structures. (**a-b**) Local structures showing the packing between Y63 and F244 in the basal state (**a**) and the break of the Y63-F244 contact in the V2Rpp-activated state (**b**). The side chain electron density of F244 is missing in the V2Rpp-bound structure and therefore not displayed. (**c-d**) Local structures showing the contact between the back loop E313 and the finger loop proximal region in the basal state (**c**) and the break of such interaction in the V2Rpp-activated state (**d**). The charged residues in the finger loop proximal region are circled by red dashed lines. The side chain electron density of E313 is missing in the V2Rpp-bound structure and therefore not displayed.

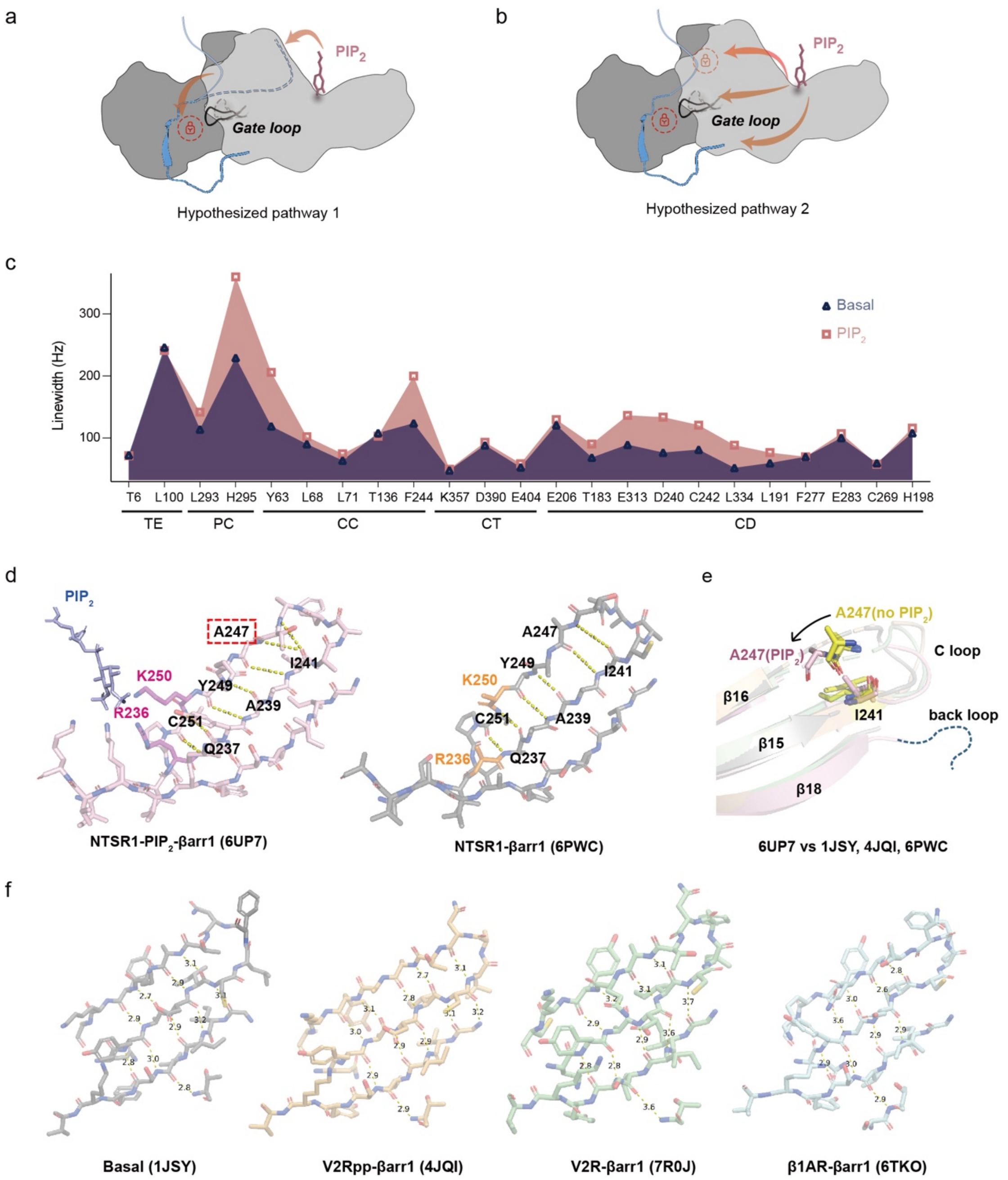

Supplementary Fig. 12. Mechanism of PIP_2 induced partial activation of βarr1. (**a-b**) Two hypothetical pathways for PIP_2 binding in the C-domain to be allosterically transduced to the gate loop. (**c**) Comparison of the NMR resonance linewidths in different structural regions in the PIP_2-bound state compared to the basal state. (**d**) Comparison of the β15-β16-β18 packing in the βarr1-NTSR1 complex structures in the presence or absence of a PIP_2 molecule. The R236 and K250 residues involved in PIP_2 binding are colored in pink in the PIP_2-bound structure (left) and in orange in the PIP_2-absent structure (right). Backbone contacts between the β15 and β16 strands are indicated. (**e**) Cartoon representation of the β15-β16-β18 local

structure depicting the movement of A147 in the presence of PIP_2 (colored in pink) compared to the other structures (colored in yellow). (**f**) The β15-β16-β18 packing in different βarr1 structures without PIP_2 are generally similar.

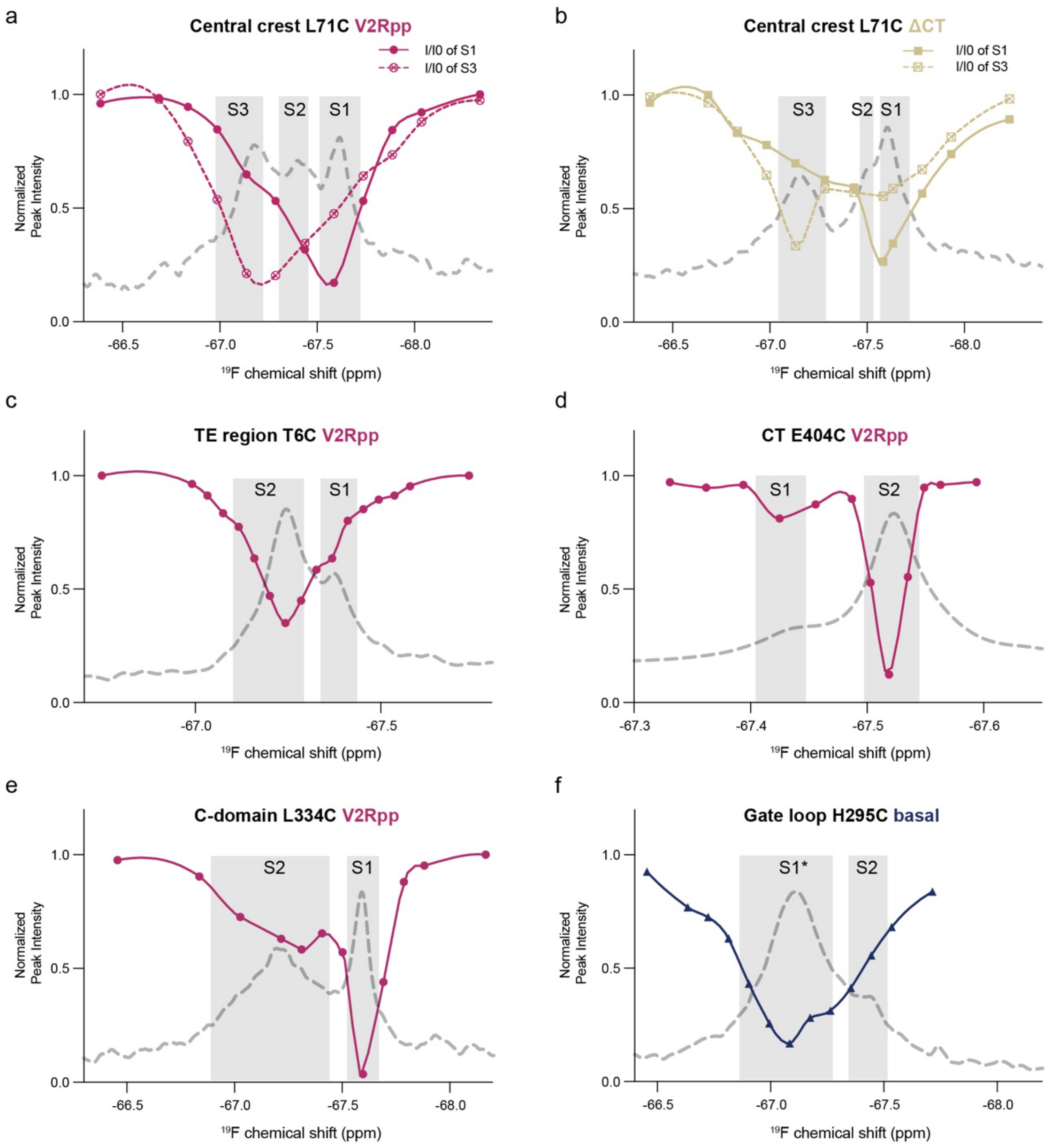

Supplementary Fig. 13. ^{19}F CEST experiments verifying conformation exchanges in βarr1. (**a-b**) ^{19}F CEST profiles of the L71C site in the V2Rpp-bound (**a**) and ΔCT states (**b**) of βarr1. In both cases, peak intensity ratios were calculated using either S1 or S3 as the reference. (**c-e**) ^{19}F CEST profiles of the V2Rpp-bound βarr1 obtained for the T6C, E404C and L334C sites. (**f**) ^{19}F CEST profiles of the gate loop H295C site obtained in the basal state. The corresponding 1D ^{19}F spectra are shown in dashed lines in all panels for comparison.

Supplementary Table 1. System setups for the MD simulations of ^{19}F-labeled βarr1

	Name	Number of atoms	Number of waters	Initial box size
1	T6C	93614	28981	86 Å×126 Å×103 Å
2	H295C	93617	28983	86 Å×126 Å×103 Å
3	L334C	94965	29433	86 Å×127 Å×103 Å
4	D390C	93591	28973	86 Å×126 Å×103 Å
5	L71C	93570	28968	86 Å×126 Å×103 Å
6	L71C-V2Rpp	81719	28981	84 Å×107 Å×110 Å

Supplementary Table 2. Summary of potential hydrogen bonds between the probe and nearby residues during the MD simulations.

#Acceptor	DonorH	Donor	Frames[1]	Frac[2]
Site 6				
6FC_6@F4	ASP_3@H	ASP_3@N	4	0.004
6FC_6@F2	ARG_103@HE	ARG_103@NE	1	0.001
6FC_6@F3	ASP_3@H	ASP_3@N	1	0.001
6FC_6@F5	ASP_3@H	ASP_3@N	1	0.001
6FC_6@N1	6FC_6@H6	6FC_6@N2	1	0.001
Site 71 (Trajectory #1: 600 ns)				
6FC_71@F1	GLY_409@H	GLY_409@N	1	0.0003
6FC_71@F2	ARG_76@HH11	ARG_76@NH1	1	0.0003
6FC_71@F4	THR_410@HG1	THR_410@OG1	1	0.0003
6FC_71@F1	LYS_160@HZ1	LYS_160@NZ	1	0.0003
6FC_71@F6	LYS_160@HZ1	LYS_160@NZ	1	0.0003
6FC_71@F4	LYS_160@HZ1	LYS_160@NZ	1	0.0003
Site 71 (Trajectory #2: 130 ns & Trajectory #3: 50 ns)				
6FC_71@*	none	none	none	none
Site 295				
6FC_295@F5	LYS_292@H	LYS_292@N	8	0.008
6FC_295@F6	LYS_292@H	LYS_292@N	7	0.007
6FC_295@F1	ARG_393@H	ARG_393@N	6	0.006
6FC_295@F4	LYS_292@H	LYS_292@N	5	0.005
6FC_295@F6	LYS_292@HZ2	LYS_292@NZ	1	0.001
6FC_295@F1	LYS_294@H	LYS_294@N	1	0.001
6FC_295@F3	ARG_393@H	ARG_393@N	1	0.001
Site 334				
6FC_334@*	none	none	none	none
Site 390				
6FC_390@N1	ALA_12@H	ALA_12@N	6	0.006
6FC_390@F6	ALA_12@H	ALA_12@N	2	0.002
6FC_390@F4	ALA_12@H	ALA_12@N	1	0.001
6FC_390@F4	LYS_10@HZ1	LYS_10@NZ	1	0.001
6FC_390@F1	ALA_12@H	ALA_12@N	1	0.001
6FC_390@F3	ALA_12@H	ALA_12@N	1	0.001
6FC_390@F2	LYS_10@HZ3	LYS_10@NZ	1	0.001
6FC_390@F5	LYS_10@HZ1	LYS_10@NZ	1	0.001
6FC_390@F4	LYS_10@HZ3	LYS_10@NZ	1	0.001

Site 71, V2Rpp-bound state				
V2Rpp_6FC_71@N1	GLY_64@H	GLY_64@N	2	0.0006
V2Rpp_6FC_71@F3	ARG_285@HE	ARG_285@NE	2	0.0006
V2Rpp_6FC_71@F5	GLU_66@H	GLU_66@N	2	0.0006
V2Rpp_6FC_71@N1	ARG_285@HE	ARG_285@NE	1	0.0003
V2Rpp_6FC_71@F4	ARG_65@HH21	ARG_65@NH2	1	0.0003
V2Rpp_6FC_71@F6	ARG_65@HH21	ARG_65@NH2	1	0.0003

[1] The total number of simulation frames in which the corresponding hydrogen bond is observed.

[2] The fraction of the number of frames in which corresponding hydrogen bond is observed compared to the total number of frames obtained in the simulations.

Supplementary Table 3. List of residues observed to have close contact with the probe and comparison of the inter-residue distances observed in experimental structures and in the simulation trajectories.

	Distance [1] (Å)				
	Contact	8AS4 [2]	1G4M	1JSY	MD distance range (min,max)
D390C	F9	7.8	7.9	7.8	(10,12)
	K10	7.3	7	7.3	(6,12)
	K11	7.2	6.9	7.2	(4,12)
	A12	9.6	9.1	9.6	(4,14)
	T19	12.1	11.7	12.1	(7,17)
L71C	R65	15.3	14.1	11.4	(8,16) [3]
	D69	9.3	8.2	8.6	(6,15)
	L73	9.2	8.1	9.3	(6,15)
	H159	16.4	17.1	13.3	(7,23)
H295C	V167	9	9.3	9	(6,10)
	R169	11.4	11.4	11.4	(9,13)
	D290	11.7	11.6	11.2	(11,15)
	G291	8	9.2	7.6	(7,12)
	K292	6	5.9	6	(4,8)
	L293	7.8	7.7	7.7	(7,12)
	D297	8.7	8.8	8.6	(7,12)
	T298	8.8	8.7	8.7	(8,12)
	R393	4.6	4.7	4.6	(4,7)
	Q394	6.2	n/a	6.6	(7,12)
	R395	6.7	n/a	6.5	(6,10)
	L396	10.4	n/a	9.2	(7,12)
T6C	V8	7.8	6	8.1	(5,12)
	R99	9.2	11.5	9.2	(4,12)
	L100	7.4	6.7	6.9	(4,12)
	R103	8.8	8.6	8.7	(4,12)
	R383	n/a	6.5	7.6	(5,20)
	L386	5.4	5.1	5.8	(4,13)
	Contact		4JQI		MD distance range (min,max)
V2Rpp-L71C	E66		12.5		(5,13)
	L68		9.8		(7,13)
	D69		7.1		(5,11)
	L73		6.9		(6,12)
	E134		8.2		(6,24)
	T136		6.5		(6,24)

[1] The distances observed in the MD simulations are calculated between the N1 atom of the probe and the Cα atom of the contacting residue. The distances observed in the experimentally-determined structures are calculated between the outmost heavy atom in the sidechain of the native residue at the labeling site and the Cα atom of the contacting residue.

[2] PDB codes for the structures used for calculation.

[3] The data for L71C is summarized from all three trajectories.

Supplementary Table 4. Primer sequences used in generating βarr1 mutants.

Primer	5'-3' sequence
Primers for generating Cys-less βarr1	
*ARRB1*_C59V_F	ACCGTCGCGTTTCGTTATGGCCGT
*ARRB1*_C59V_R	AAACGCGACGGTCAGCGTCACATACACAC
*ARRB1*_ C125S_F	GCCGGCTTCTGTGACCCTGCAGC
*ARRB1*_C125S_R	CAGAAGCCGGCAGATTCGGCGG
*ARRB1*_C140L_F	GCTCTGGGCGTGGATTATGAAGTGA
*ARRB1*_C140L_R	CGCCCAGAGCTTTGCCAGTATCTTCCG
*ARRB1*_C150V_F	GCGTTTGTCGCAGAAAATCTGGAAGAAAAGATTC
*ARRB1*_C150V_R	TGCGACAAACGCCTTCACTTCATAATCC
*ARRB1*_C242V_F	GGATATTGTCCTGTTTAATACCGCGCAGT
*ARRB1*_C242V_R	ACAGGACAATATCCGCATATTGACGCAC
*ARRB1*_C251V_F	AAAGTCCCCGTGGCTATGGAGG
*ARRB1*_C251V_R	CACGGGGACTTTATACTGCGCGGTATTAAACAG
*ARRB1*_C269S_F	CGTTTTCAAAGGTGTATACCCTGACTCCG
*ARRB1*_C269S_R	CACCTTTGAAAACGTGGAGCTCGGAG
Primers for introducing single-site Cys mutation in βarr1	
*ARRB1*_T6C_F	GATGCCGTGTGTTCAAGAAGGCG
*ARRB1*_T6C_R	ACACACGGCATCCTTTGTCGCCCATGC
*ARRB1*_Y63C_F	GTTGCGGCCGTGAAGATTTGGATG
*ARRB1*_Y63C_R	CGGCCGCAACGAAACGCGACGGTC
*ARRB1*_L68C_F	GATTGCGATGTGCTGGGCTTGACC
*ARRB1*_L68C_R	GCACATCGCAATCTTCACGGCCATAACGAA
*ARRB1*_L71C_F	TGTGCGGCTTGACCTTTCGTAAAGA
*ARRB1*_L71C_R	TCAAGCCGCACACATCCAAATCTTCACGGC
*ARRB1*_L100C_F	TCGTTGCCAGGAGCGTCTGATTAAGAA
*ARRB1*_L100C_R	TCCTGGCAACGAGTCAGCGGCTTCT
*ARRB1*_T136C_F	GATTGCGGCAAAGCTCTGGGCG
*ARRB1*_T136C_R	CTTTGCCGCAATCTTCCGGCCCTGGC
*ARRB1*_T183C_F	AGCCGTGCGCGGAAACCACGCG
*ARRB1*_T183C_R	GCGCACGGCTGCGGACCCG
*ARRB1*_L191C_R	CGCTCATGCAGAACTGACGCGTGGTTTCC
*ARRB1*_L191C_F	TCTGCATGAGCGATAAACCGCTGC
*ARRB1*_H198C_F	TGTGCCTGGAAGCAAGCCTGGA
*ARRB1*_H198C_R	CTTCCAGGCACAGCGGTTTATCGCTCATCA
*ARRB1*_E206C_F	GGATAAATGCATTTACTACCATGGCGAACC
*ARRB1*_E206C_R	GTAAATGCATTTATCCAGGCTTGCTTCCA

*ARRB1*_D240C_F	CGTGCATTGTCCTGTTTAATACCGCGC
*ARRB1*_D240C_R	GGACAATGCACGCATATTGACGCACGCTG
*ARRB1*_F244C_F	CCTGTGCAATACCGCGCAGTATAAAGTCCC
*ARRB1*_F244C_R	GGTATTGCACAGGACAATATCCGCATATTGACGC
*ARRB1*_F277C_R	TAAGCACGGAGTCAGGGTATACACC
*ARRB1*_F277C_F	GACTCCGTGCTTAGCGAATAATCGGGAAAAGCGT
*ARRB1*_E283C_F	CGGTGCAAGCGTGGCCTGGCGC
*ARRB1*_E283C_R	CGCTTGCACCGATTATTCGCTAAAAACGGAGT
*ARRB1*_L293C_F	GGGAAGTGCAAGCACGAAGACACCAATCTGG
*ARRB1*_L293C_R	CTTGCACTTCCCATCAAGCGCCAGG
*ARRB1*_H295C_F	GAAGTGCGAAGACACCAATCTGGCTA
*ARRB1*_H295C_R	GTCTTCGCACTTCAGCTTCCCATCAAGCG
*ARRB1*_E313C_F	CGTTGCATTCTGGGCATTATAGTGAGC
*ARRB1*_E313C_R	CCAGAATGCAACGATTGGCTCCTTCACG
*ARRB1*_E334C_R	CCAGGCAGCCACCACGGCTCAC
*ARRB1*_E334C_F	GGCTGCCTGGGGGATCTGGCG
*ARRB1*_K357C_R	TTCGCACGGTTTCGGATGCATAAGGG
*ARRB1*_K357C_F	GAAACCGTGCGAAGAACCACCGCATCGGGA
*ARRB1*_N375C_R	CAATCAGGCACGTGTCCACCGGGGTCTCAT
*ARRB1*_N375C_F	CGTGCCTGATTGAACTGGATACCAATGACG
*ARRB1*_F388C_F	TAGTGTGCGAAGACTTTGCGCGGC
*ARRB1*_F388C_R	GTCTTCGCACACTATATCATCGTCATTGGTATCC
*ARRB1*_D390C_F	CGAATGCTTTGCGCGGCAGCG
*ARRB1*_D390C_R	CGCAAAGCATTCGAACACTATATCATCGTCATTG
*ARRB1*_M399C_F	GGGTGCAAAGATGACAAAGAGGAAGAGG
*ARRB1*_M399C_R	CATCTTTGCACCCTTTCAGGCGCTGC
*ARRB1*_E404C_F	TGACAAATGCGAAGAGGAGGATGGCAC
*ARRB1*_E404C_R	CTTCGCATTTGTCATCTTTCATCCCTTTCAG
Primers for generating truncation mutants of βarr1	
*ARRB1*_1-382_F	CCAATTGACTCGAGCACCACC
*ARRB1*_1-382_R	CTCGAGTCAATTGGTATCCAGTTCAATCAGG
*ARRB1*_1-395_F	GCGCTGACTCGAGCACCACC
*ARRB1*_1-395_R	CGAGTCAGCGCTGCCGCGCAAA
Primers for the 3Q-mutant of βarr1	
*ARRB1*_K232Q_F	TCCAGATCAGCGTGCGTCAATATGCG
*ARRB1*_K232Q_R	CGCTGATCTGGATCTTTTTCACTGTTTTGTTGGTG
*ARRB1*_R236Q_F	AGCGTGCAGCAATATGCGGATATTGTCCTG
*ARRB1*_R236Q_R	TTGCTGCACGCTGATCTTGATCTTTTTCAC

*ARRB1*_K250Q_F	TATCAGGTCCCCGTGGCTATGG
*ARRB1*_K250Q_R	GGGGACCTGATACTGCGCGGTATTAAACAG

Supplementary Discussion

MD simulations of ^{19}F-labeled βarr1

To support that introduction of the wPSP-6F label does not cause artificial contacts and perturb βarr1 local structures, we performed MD simulation of apo-βarr1 labeled at the T6C, L71C, H295C, L334C and D390C sites, as well as V2Rpp-bound βarr1 labeled at the L71C site. As summarized in Supplementary Table 2, potential hydrogen bonds are sporadically detected in a very limited number of frames in the simulation trajectories of all the labeling sites, and none of them are observed to stably exist. Furthermore, we calculated all close contacts (defined as the situation where any two carbon atoms from two residues are within 7 Å distance) observed in the simulation trajectories. In Supplementary Table 3, we list all residues (excluding residues at the +1 and -1 positions of the labeling site) that are observed to form close contacts with the labeled probe during the MD. To evaluate whether these contacts deviate from the native conformation of βarr1, we compared the distances observed in the available crystal or cryo-EM structures with the MD data (Supplementary Table 3). The results suggest that the distances between the specific residue pair in the native βarr1 structure fall within the distance range detected in the MD simulation. By plotting the locations of the probe sidechain in all simulation trajectories onto the βarr1 structure (Supplementary Fig. 5), we can see that the probe sidechain is uniformly distributed in a continuous space allowed by the local structure, and observe no apparent bias towards specific orientations. For labeling sites that are more exposed (e.g. L71C and L334C), we observe that the probe sidechain is flexible and samples a wide range of orientations. Therefore, the contacts detected between the probe with nearby residues are most probably the results of fast sidechain motions. Moreover, three independent simulation trajectories (lasting 600 ns, 130 ns and 50 ns) of the L71C-labeled sample starting with different probe sidechain orientations suggested similar results. Taken together, the MD results suggest that the wPSP-6F probe is not likely to introduce artificial contacts with nearby residues, which is in agreement with our results from ^{1}H NMR and CD spectroscopy, as well as the clathrin and Fab30 binding assays (Supplementary Fig. 2-

4). Nevertheless, the current simulations are performed in the range of several hundreds of nanoseconds, which is sufficient for monitoring the probe sidechain motions, but we cannot exclude the possibility that the labeling may affect the conformational landscape of βarr1 on longer timescales.

Conformational dynamics in the gate loop

Although both the L293C and H295C sites in the gate loop show two peaks in the basal state, their spectral changes upon CT-removal or V2Rpp-binding are somewhat different. Here, we try to provide a possible explanation of these differences.

In the inactive state structures, H295 packs closely with R393 from the CT, whereas release of the CT removes such constraint and allows the H295 side chain to flip up into a conformation in which it does not form obvious contacts with surrounding residues (Supplementary Fig. 8). Therefore, during the gate loop activation, H295 transits from a structurally stabilized (or locked) conformation into a relatively free one. The NMR spectra changes at the H295C site match this structural change, displaying a single S2 peak in the activated states with significantly decreased linewidths.

Interpretation of the spectral changes for the L293C site is more difficult. Dual conformations are observed in all three states, suggesting this site may intrinsically adopt two alternative conformations. In the basal state, the S1 peak is dominant, occupying over 80% of the population. CT release results in the decrease of chemical shift difference between the two peaks, which may be attributed to a faster exchange rate or a more similar chemical environment between the two conformations. Binding to V2Rpp leads to the separation of the two peaks (S1' and S2') again, but with slightly altered chemical shift values (which may reflect subtle variations in local environments) compared to the basal state, and a similar population distribution as in the ΔCT state. In both the ΔCT and V2Rpp-bound states, the population of the S2 resonance increases to close to 30%. In the inactive βarr1 structures, the L293 side chain points rightward to contact the neighboring loop (N281-G286), and in the crystal structure of the V2Rpp-bound βarr1 and that of the CT-truncated variant of visual arrestin (the pre-activated state), the gate loop is stabilized into a conformation with the L293 (I293 in the visual

arrestin) side chain pointing leftward to contact the β2-β3 loop (Supplementary Fig. 8). We speculate that the S1 and S2 (or the S1' and S2') peaks of L293C may correspond to the rightward-facing and leftward-facing conformations respectively, however, they may also originate from alternative conformations different from those observed in the crystal structures.

Unlike the H295C site, the S2' population of L293C in the V2Rpp-bound state is elevated but still not dominant, the reason for which is unclear. In a previous molecular dynamics simulation study of βarr1 activation by Latorraca *et al.* [3], it was shown that in the V2Rpp-bound state, different phosphorylation sites could compete in engaging with the K294 residue in the gate loop, leading to activating (e.g. pT360) or deactivating (e.g. pS357) outcomes, implying complexity of the gate loop conformational dynamics. Thus, in the V2Rpp-bound state of βarr1, K294 is likely to adopt alternate conformations that either promote or inhibit activation, and may pull the neighboring L293 residue into differently stabilized conformations. This provides one possible explanation for the spectral complexity of L293C. Alternatively, it is also possible that spin labeling at the L293C site more significantly changes the local dynamics.

It should be cautioned that mutation and labeling would inevitably affect the local dynamics, and therefore absolute values (e.g., chemical shifts, populations, *etc.*) are expected to be different from the wild-type protein. Nevertheless, spectral differences between the three functional states clearly reveal that βarr1 activation promotes the S2 conformation for both L293C and H295C labeling sites.

Supplementary References

1 Chai, Z. *et al.* Visualizing proteins in human cells at near-physiological concentrations with sensitive (19)F NMR chemical tags. *Angew. Chem. Int. Ed. Engl.* **62**, e202300318 (2023).

2 Becker, W., Bhattiprolu, K. C., Gubensäk, N. & Zangger, K. Investigating protein-ligand interactions by solution nuclear magnetic resonance spectroscopy. *Chemphyschem* **19**, 895-906 (2018).

3 Latorraca, N. R. *et al.* How GPCR phosphorylation patterns orchestrate arrestin-mediated signaling. *Cell* **183**, 1813-1825 (2020).
