# Supplementary material for: Distinct activation mechanisms of β-arrestin-1 revealed by 19F NMR spectroscopy
Source: Nat Commun. 2023 Nov 29;14:7865. doi: 10.1038/s41467-023-43694-1 (PMC10686989; doi:10.1038/s41467-023-43694-1)

Time course of the backbone heavy atom root mean square deviation (RMSD) from the initial structure of all residues in βarr1 labeled with wPSP-6F at different sites

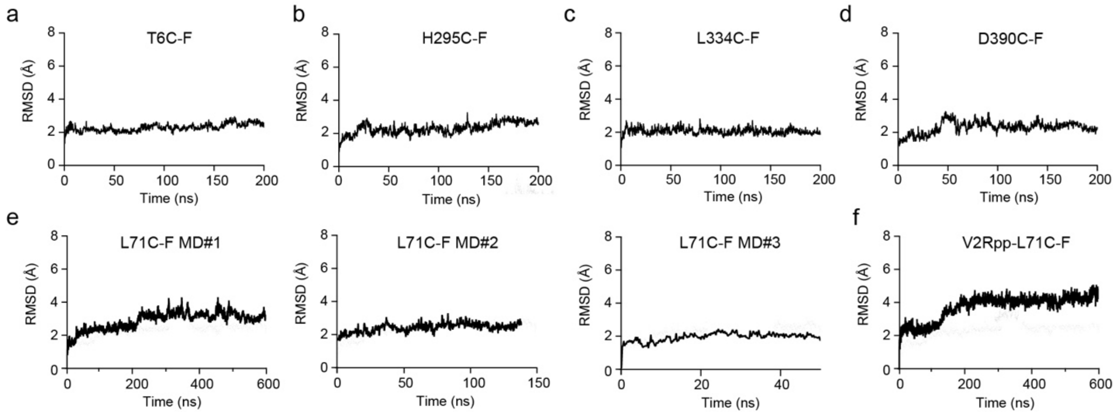

Supplement: Supplementary file 3 — Supplementary Data 1 [file 41467_2023_43694_MOESM3_ESM.zip › Supplementary-Data-1/RMSD.pdf]
