# Supplementary material for: Distinct activation mechanisms of β-arrestin-1 revealed by 19F NMR spectroscopy
Source: Nat Commun. 2023 Nov 29;14:7865. doi: 10.1038/s41467-023-43694-1 (PMC10686989; doi:10.1038/s41467-023-43694-1)

RESP atomic charges of Modified residue 6FC:

Atom name	RESP charge
C1	0.448008
O1	-0.51616
C2	-0.46393
S1	-0.13341
H1	0.075416
H2	0.221313
H3	0.221313
C3	0.261777
C4	-0.38687
C5	-0.38687
C6	0.410937
H4	0.204581
C7	0.410937
H5	0.204581
N1	-0.57129
C8	0.482078
C9	0.482078
F1	-0.18286
F2	-0.18286
F3	-0.18286
F4	-0.18286
F5	-0.18286
F6	-0.18286
N2	-1.05551
H6	0.408215
H7	0.408215
C10	0.538351
O2	-0.65105
H8	0.484454

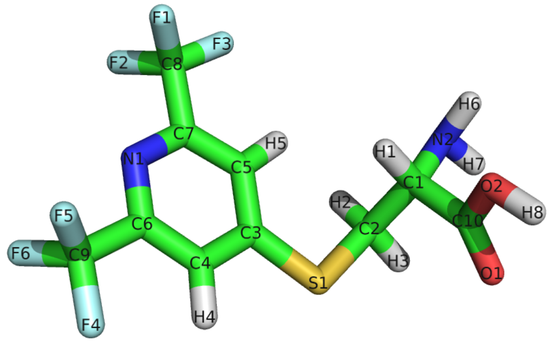

Supplement: Supplementary file 3 — Supplementary Data 1 [file 41467_2023_43694_MOESM3_ESM.zip › Supplementary-Data-1/RESP-6FC.pdf]
