# Supplementary material for: Distinct activation mechanisms of β-arrestin-1 revealed by 19F NMR spectroscopy
Source: Nat Commun. 2023 Nov 29;14:7865. doi: 10.1038/s41467-023-43694-1 (PMC10686989; doi:10.1038/s41467-023-43694-1)
Supplement: Supplementary file 4 — Reporting Summary [file 41467_2023_43694_MOESM4_ESM.pdf]

nature portfolio

Corresponding author(s): Yunfei Hu, Changwen Jin

Last updated by author(s): 11/13/2023

Reporting Summary

Nature Portfolio wishes to improve the reproducibility of the work that we publish. This form provides structure for consistency and transparency in reporting. For further information on Nature Portfolio policies, see our Editorial Policies and the Editorial Policy Checklist.

Statistics

For all statistical analyses, confirm that the following items are present in the figure legend, table legend, main text, or Methods section.

n/a	Confirmed	
☐	☒	The exact sample size (*n*) for each experimental group/condition, given as a discrete number and unit of measurement
☐	☒	A statement on whether measurements were taken from distinct samples or whether the same sample was measured repeatedly
☒	☐	The statistical test(s) used AND whether they are one- or two-sided<br>*Only common tests should be described solely by name; describe more complex techniques in the Methods section.*
☒	☐	A description of all covariates tested
☒	☐	A description of any assumptions or corrections, such as tests of normality and adjustment for multiple comparisons
☐	☒	A full description of the statistical parameters including central tendency (e.g. means) or other basic estimates (e.g. regression coefficient) AND variation (e.g. standard deviation) or associated estimates of uncertainty (e.g. confidence intervals)
☒	☐	For null hypothesis testing, the test statistic (e.g. *F*, *t*, *r*) with confidence intervals, effect sizes, degrees of freedom and *P* value noted<br>*Give P values as exact values whenever suitable.*
☒	☐	For Bayesian analysis, information on the choice of priors and Markov chain Monte Carlo settings
☒	☐	For hierarchical and complex designs, identification of the appropriate level for tests and full reporting of outcomes
☒	☐	Estimates of effect sizes (e.g. Cohen's *d*, Pearson's *r*), indicating how they were calculated

Our web collection on statistics for biologists contains articles on many of the points above.

Software and code

Policy information about availability of computer code

Data collection	For NMR data collection, Bruker Topspin 4.2.0 was used. For molecular simulation, GROMACS 2020.3, AMBER 18, Rosetta 3.12, Gaussian 09 and PyMol 2.5.2 were used. The AMBER force field ff14SB for proteins was used.
Data analysis	Bruker Topspin Software 4.2.0 and MestReNova 12.0.0 was used for NMR data processing and analysis. GraphPad Prism® 9, MATLAB R2020 and PyMol 2.5.2 was used for data and structural analysis. Molecular simulation data were analyzed with AMBER 18 and CHARMM-gui.

For manuscripts utilizing custom algorithms or software that are central to the research but not yet described in published literature, software must be made available to editors and reviewers. We strongly encourage code deposition in a community repository (e.g. GitHub). See the Nature Portfolio guidelines for submitting code & software for further information.

Data

Policy information about availability of data

All manuscripts must include a data availability statement. This statement should provide the following information, where applicable:

- Accession codes, unique identifiers, or web links for publicly available datasets
- A description of any restrictions on data availability
- For clinical datasets or third party data, please ensure that the statement adheres to our policy

Source data are provided as a source data file. The NMR chemical shifts and linewidths data are made available through the public repository Zenodo. The MD simulation parameter files are provided in Supplementary Data 1 file. The previously published structures used in this study are available in the PDB database under accession codes 1JSY, 6UP7, 6PWC, and 7DFA. Information on how to access the files are provided in the main text in the data availability section.

Research involving human participants, their data, or biological material

Policy information about studies with human participants or human data. See also policy information about sex, gender (identity/presentation), and sexual orientation and race, ethnicity and racism.

Reporting on sex and gender	N/A
Reporting on race, ethnicity, or other socially relevant groupings	N/A
Population characteristics	N/A
Recruitment	N/A
Ethics oversight	N/A

Note that full information on the approval of the study protocol must also be provided in the manuscript.

Field-specific reporting

Please select the one below that is the best fit for your research. If you are not sure, read the appropriate sections before making your selection.

☒ Life sciences ☐ Behavioural & social sciences ☐ Ecological, evolutionary & environmental sciences

For a reference copy of the document with all sections, see nature.com/documents/nr-reporting-summary-flat.pdf

Life sciences study design

All studies must disclose on these points even when the disclosure is negative.

Sample size	No sample size calculations were performed. Sample size was determined to be adequate based on the magnitude and consistency of measurable differences between groups.
Data exclusions	No data were excluded from analysis.
Replication	The NMR data of all labeling sites in basal state were collected three times using different batches of protein samples, and the results were replicable. The NMR data of the V2Rpp-bound state (sites 6, 63, 68, 71, 206, 244, 277, 295, 357, 375, 390 and 404) and PIP2-bound states (sites 6, 71, 295, 334 and 390) were collected twice using different batches of protein samples, and the results were replicable. NMR titration of V2Rpp binding experiment was repeated twice, and the results were replicable.
Randomization	NMR experiments were systematically conducted with appropriate controls, and no randomization is involved.
Blinding	No blinding was attempted or needed. There was no group allocation performed in this study.

Reporting for specific materials, systems and methods

We require information from authors about some types of materials, experimental systems and methods used in many studies. Here, indicate whether each material, system or method listed is relevant to your study. If you are not sure if a list item applies to your research, read the appropriate section before selecting a response.

Materials & experimental systems

n/a	Involved in the study
x	Antibodies
x	Eukaryotic cell lines
x	Palaeontology and archaeology
x	Animals and other organisms
x	Clinical data
x	Dual use research of concern
x	Plants

Methods

n/a	Involved in the study
x	ChIP-seq
x	Flow cytometry
x	MRI-based neuroimaging
